# Transportin-SR Is Required for Proper Splicing of *Resistance* Genes and Plant Immunity

**DOI:** 10.1371/journal.pgen.1002159

**Published:** 2011-06-30

**Authors:** Shaohua Xu, Zhibin Zhang, Beibei Jing, Patrick Gannon, Jinmei Ding, Fang Xu, Xin Li, Yuelin Zhang

**Affiliations:** 1 Graduate Program in Chinese Academy of Medical Sciences and Peking Union Medical College, Beijing, China; 2 National Institute of Biological Sciences, Beijing, China; 3 Michael Smith Laboratories, University of British Columbia, Vancouver, Canada; University of California Davis Genome Center, United States of America

## Abstract

Transportin-SR (TRN-SR) is a member of the importin-β super-family that functions as the nuclear import receptor for serine-arginine rich (SR) proteins, which play diverse roles in RNA metabolism. Here we report the identification and cloning of *mos14* (*modifier of snc1-1*, *14*), a mutation that suppresses the immune responses conditioned by the auto-activated Resistance (R) protein snc1 (suppressor of npr1-1, constitutive 1). *MOS14* encodes a nuclear protein with high similarity to previously characterized TRN-SR proteins in animals. Yeast two-hybrid assays showed that MOS14 interacts with AtRAN1 via its N-terminus and SR proteins via its C-terminus. In *mos14-1*, localization of several SR proteins to the nucleus was impaired, confirming that MOS14 functions as a TRN-SR. The *mos14-1* mutation results in altered splicing patterns of *SNC1* and another *R* gene *RPS4* and compromised resistance mediated by *snc1* and *RPS4*, suggesting that nuclear import of SR proteins by MOS14 is required for proper splicing of these two *R* genes and is important for their functions in plant immunity.

## Author Summary

Plant immune receptors encoded by *Resistance* (*R*) genes play essential roles in defense against pathogens. Multiple *R* genes are alternatively spliced. How plants regulate the splicing of these *R* genes is unclear. In this study, we identified MOS14 as an important regulator of two *R* genes, *SNC1* and *RPS4*. Further analysis showed that MOS14 functions as the nuclear import receptor for serine-arginine rich (SR) proteins, which play diverse roles in RNA metabolism. Loss of the function of MOS14 results in altered splicing patterns of *SNC1* and *RPS4* and compromised resistance mediated by *snc1* and *RPS4*, suggesting that nuclear import of SR proteins by MOS14 is required for proper splicing of these two *R* genes and is important for their functions in plant immunity.

## Introduction

In eukaryotes, the nuclear envelope forms a barrier between the cytoplasm and the nucleus. Trafficking of macromolecules across the nuclear envelope occurs through the nuclear pore complex (NPC) [1]. Previous studies on MOS3 [2], MOS6 [3], MOS7 [4] and MOS11 [5] have revealed the importance of nucleocytoplasmic trafficking in plant immunity. Mutations in *MOS3*, *MOS6*, *MOS7* and *MOS11* suppress the constitutive defense responses of *snc1* (*suppressor of npr1-1*, *constitutive 1*), a gain-of-function mutant carrying a mutation in a Toll/interleukin-1 receptor-Nucleotide Binding-Leucine Rich Repeat (TIR-NB-LRR) R protein [6]. *MOS3* encodes the nucleoporin Nup96 [2], whereas *MOS11* encodes a putative RNA binding protein [5]. Both MOS3 and MOS11 are required for mRNA export. *MOS6* encodes a putative importin-α [3], whereas *MOS7* encodes another nucleoporin, Nup88, which is required for nuclear accumulation of snc1 and two general defense regulators, Enhanced Disease Susceptibility 1 (EDS1) and Nonexpresser of PR genes 1 (NPR1) [4].

Nuclear import receptors play essential roles in transferring proteins from the cytoplasm to the nucleus. The largest group of nuclear import receptors belong to the importin-β super-family. Members of the importin-β super-family have rather low overall sequence similarity but they all have a conserved N-terminal RAN-binding domain [7], [8]. The import receptors recognize the nuclear localization sequence (NLS) of target proteins to facilitate their transport through the NPC. Upon RAN-GTP binding to importin-β, the importin-β complex is dissociated and the cargo is released into the nucleus.

The importin-β super-family can be divided into several sub-families according to the direction and the cargo type they transport [9]. Among them, the transportin-SR (TRN-SR) subfamily functions as nuclear import receptors for serine-arginine rich (SR) proteins. TRN-SR was originally identified as an interactor of SR domains of ASF/SF2 [10] and papillomavirus E2 [11]. In humans, the C-terminus of TRN-SR interacts with SR proteins and the interaction can be disrupted upon RAN-binding to its N-terminus [10].

SR proteins are a highly conserved family of nuclear proteins that play important roles in splicing [12]–[14]. They contain RNA recognition motifs (RRM) at the N-terminus and an arginine-serine rich (RS) domain at the C-terminus. The NLS is located in the RS domain. SR proteins not only function as splicing factors for constitutive splicing [15], [16], they also regulate alternative splicing through splice site selection in a concentration-dependent manner [17], [18].

Several plant *R* genes including the tobacco *N* gene [19], the barley *Mla6* [20], Arabidopsis *SNC1* [21] and *RPS4* [22]–[24] are alternatively spliced. For example, six transcript variants (TV) have been identified for *RPS4* [23], [24]. Compromised *RPS4*-mediated resistance resulting from a lack of the TVs suggests that alternative splicing of *RPS4* is required for its function [23]. However, it is unclear how alternative splicing of these *R* genes is controlled and why it is necessary for immunity. In this study, we report that Arabidopsis *MOS14* encodes a TRN-SR that is required for proper splicing of *SNC1* and *RPS4*, suggesting that SR proteins may play important roles in the control of the splicing of these two *R* genes.

## Results

### Identification of *mos14-1 snc1 npr1*

**Figure 1:**
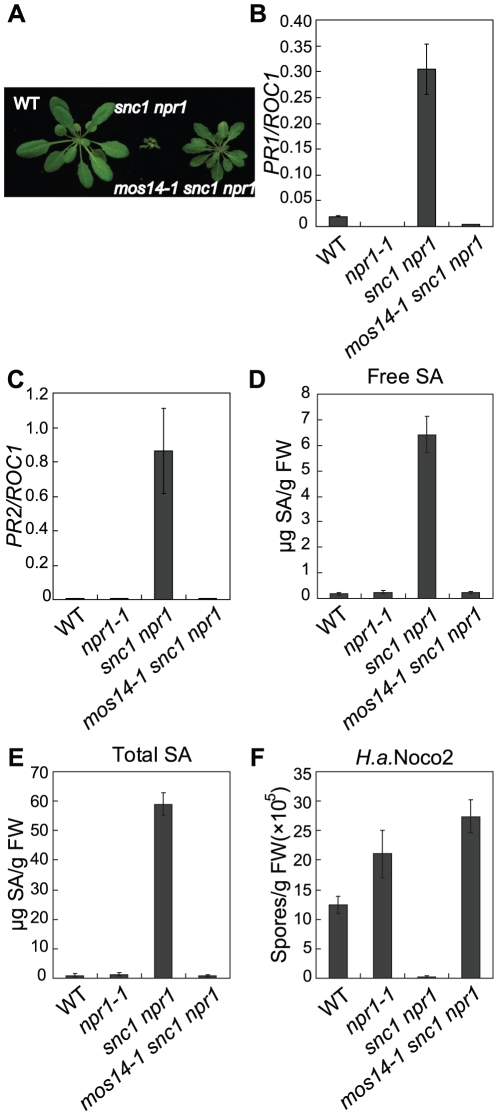
Constitutive immune responses in *snc1* are suppressed by *mos14-1*. (A) Morphology of five-week-old soil-grown plants of the indicated genotypes. (B–C) *PR1* (B) or *PR2* (C) expression in the indicated genotypes. RNAs were extracted from two-week-old plants grown on MS media and reverse transcribed to obtain cDNA for real-time PCR analysis. Values were normalized to the expression of the reference gene *Cyclophilin* (*ROC1*). Error bars represent ±SD of three replicates. (D–E) Free SA (D) and total SA (E) levels in the indicated genotypes. SA was extracted from five-week-old soil-grown plants and measured by HPLC. Error bars represent ±SD of three replicates. (F) Growth of *H. a.* Noco2 on the indicated genotypes. Plants were inoculated by *H. a.* Noco2 at 5×10^4^ spores/ml and spores were collected and counted seven days later. Error bars represent ±SD of three replicates.

Arabidopsis *snc1* constitutively activates defense responses and displays enhanced resistance to pathogens. *snc1* mutant plants exhibit dwarf morphology with curly leaves. Suppressor screens of *snc1* have previously been carried out using fast neutron and T-DNA insertional mutagenesis [2], [25]. To identify additional suppressor mutants of *snc1*, we treated *snc1 npr1* seeds with ethane methyl sulfonate (EMS) and screened the M2 plants for mutants that suppressed *snc1* dwarfism. From this population, we identified *mos14-1 snc1 npr1* (Figure 1A).

In *snc1 npr1*, defense marker gene *PR1* and *PR2* are constitutively expressed. As shown in Figure 1B and 1C, constitutive activation of *PR1* and *PR2* is suppressed in *mos14-1 snc1 npr1*. Analysis of SA levels also showed that the elevated SA levels in *snc1 npr1* are suppressed by *mos14-1* (Figure 1D and 1E). To test whether enhanced pathogen resistance in *snc1 npr1* is affected by *mos14-1*, *mos14-1 snc1 npr1* seedlings were challenged with the virulent oomycete pathogen *Hyaloperonospora arabidopsidis* (*H.a*.) Noco2. As shown in Figure 1F, resistance to *H. a.* Noco2 is lost in *mos14-1 snc1 npr1*.

### Map-based cloning of *mos14-1*

**Figure 2:**
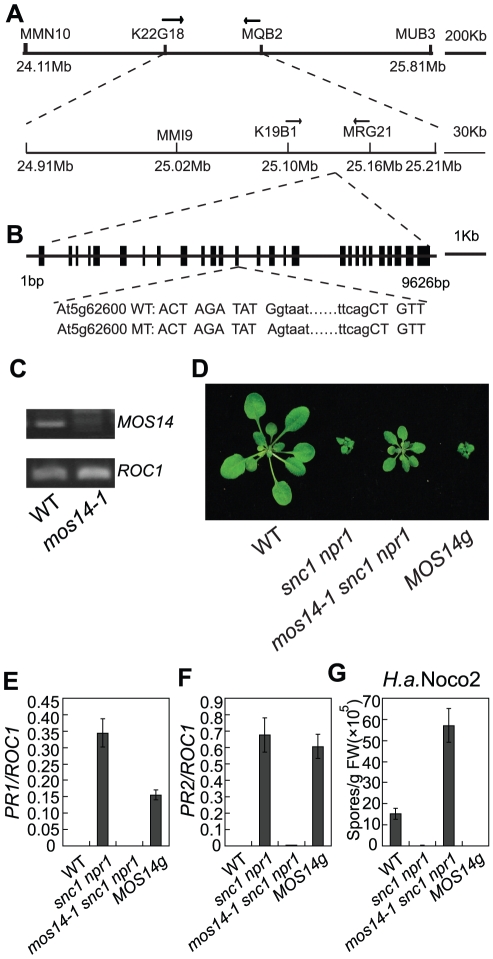
Map-based cloning of *mos14-1*. (A) Mapping of *mos14-1*. BAC clones and markers are indicated. The *mos14-1* mutation is flanked between markers K19B1 and MRG21. (B) Gene structure of *MOS14* and the mutation site in *mos14-1*. The exons are indicated with boxes and introns with lines. The mutation site is located at the junction between the 13th intron and 13th exon. The lower case letters represent nucleotides in the intron and the uppercase letters represent nucleotides in the exon. (C) Expression of *MOS14* in *mos14-1*. Primers (F47 and SNPR, Table S1) flanking the mutation site were used to amplify *MOS14* from wild type and *mos14-1* cDNA. *ROC1* was used as loading control. (D) Morphology of *mos14-1 snc1 npr1* carrying the *MOS14* transgene. Five-week-old soil-grown plants were photographed. “*MOS14g*” stands for “*mos14-1 snc1 npr1* containing the *MOS14* transgene under its native promoter”. (E–F) Restoration of *PR1* (E) and *PR2* (F) expression in *mos14-1 snc1 npr1* by the *MOS14* transgene. (G) Restoration of resistance to *H. a.* Noco2 in *mos14-1 snc1 npr1* by the *MOS14* transgene.

To map the *mos14-1* mutation, we crossed *mos14-1 snc1 npr1* (in the Columbiaecotype background) with Landsberg *erecta* (Ler)-*snc1* [2]. In the F2 mapping population, about a quarter of the progeny showed morphology similar to the triple mutant. Crude mapping using 24 F2 plants revealed that *mos14-1* is linked to the lower arm of chromosome 5 (Figure 2A). Further analysis indicated that *mos14-1* is flanked by marker MMN10 and MUB3. Fine mapping using about 1200 F2 plants narrowed *mos14-1* to a 60 kb region between marker K19B1 and MRG21. To identify the *mos14-1* mutation, PCR fragments covering this 60 kb region was amplified directly from *mos14-1 snc1 npr1* and sequenced. A single G to A mutation was found in *At5g62600* (Figure 2B), which is located at the junction of the 13th intron and 13th exon of the gene. RT-PCR analysis using primers flanking the mutation showed that splicing of *At5g62600* was affected by the mutation (Figure 2C). The RT-PCR fragments were cloned into the pGEM-T vector. Subsequent sequence analysis of cDNA clones from *mos14-1* revealed that they fell into six different classes. All of them represent transcript variants that were incorrectly spliced. An alignment of wild type cDNA and the cDNA variants from *mos14-1* are shown in Figure S1.

To confirm that the mutation in *At5g62600* is responsible for the suppression of *snc1 npr1* mutant phenotypes, a genomic clone containing *At5g62600* was constructed and transformed into *mos14-1 snc1 npr1*. Transgenic plants from five independent lines carrying the wild type *At5g62600* displayed *snc1*-like morphology (Figure 2D). Further analysis of a representative transgenic line showed that the expression of *PR1* and *PR2* was similar to *snc1 npr1* (Figure 2E and 2F). In addition, resistance to *H. a.* Noco2 was also restored in the transgenic line (Figure 2G), confirming that *At5g62600* complemented *mos14-1* and *MOS14* is *At5g62600*.

To obtain the *mos14-1* single mutant, we backcrossed *mos14-1 snc1 npr1* with wild type plants. The *mos14-1* single mutant was obtained by genotyping the F2 plants. The *mos14-1* single mutant flowers late and has reduced fertility. Besides, it exhibits small stature (Figure S2). When the genomic clone of *At5g62600* was introduced into the *mos14-1* single mutants, it reverted the size and fertility of the mutant to wild type-like and also suppressed the late flowering phenotype, showing that the developmental phenotypes observed in *mos14-1* are caused by the *mos14-1* mutation.

### *MOS14* encodes a transporter for SR proteins

*MOS14* is a single copy gene in Arabidopsis. It encodes a protein with 25% identity and 45% similarity to the TRN-SR in *Drosophila*, suggesting that MOS14 may be a transporter for SR proteins. MOS14 and its animal homologs are highly conserved at their N-terminus (Figure S3), which contain the importin-β N-terminal domains.

**Figure 3:**
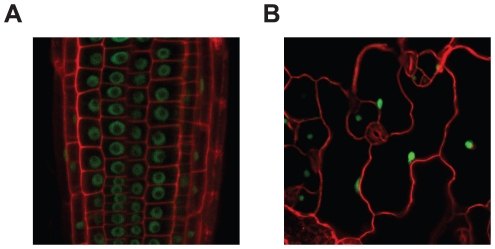
MOS14 localizes to the nucleus. GFP fluorescence in root (A) and epidermal (B) cells from transgenic plants expressing MOS14-GFP under its own promoter in Col-0 (WT). Cell walls were stained with PI before confocal microscopy, which fluoresces red.

To determine the subcellular localization of MOS14, transgenic plants expressing MOS14 under its native promoter with a C-terminal GFP tag were generated in both wild type and *mos14-1* backgrounds. Expression of MOS14-GFP in *mos14-1* suppresses the developmental phenotypes of *mos14-1* (Figure S4), suggesting that the fusion protein is functional. Confocal fluorescence microscopy analysis of transgenic plants expressing the MOS14-GFP fusion protein showed that the GFP signal is found exclusively in the nucleus (Figure 3), indicating that MOS14 is a nuclear protein. In the nuclei of root cells, GFP fluorescence was excluded from a large part of the nucleus, probably the nucleolus. We did not observe similar exclusion of MOS14-GFP from parts of the nuclei in epidermal cells, probably because these nuclei are much smaller than those in root cells.

**Figure 4:**
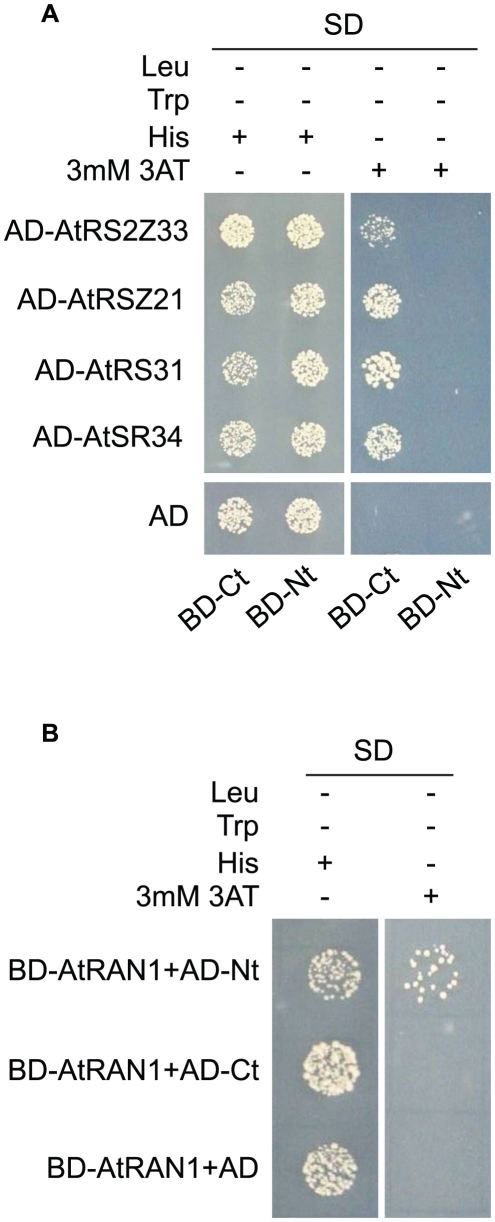
MOS14 interacts with AtRAN1 and SR proteins in yeast two-hybrid assays. (A) Interaction between the C-terminus (Ct, amino acid 282–958) of MOS14 and the indicated SR proteins. (B) Interaction between the N-terminus (Nt, amino acid 1–281) of MOS14 and AtRAN1. 3 mM 3-AT was included in the medium to increase the stringency of selection. SD, synthetic dropout medium.

In animals, TRN-SR binds SR proteins via its C-terminus and transport SR proteins through the nuclear envelope. Binding of RAN-GTP to the N-terminus of TRN-SR in nucleus results in the release of SR proteins. To test whether MOS14 is able to interact with SR proteins, the N-terminus (1–281) and C-terminus (282–958) of MOS14 were expressed in the bait vector and four selected Arabidopsis SR proteins (AtRS2Z33, AtRSZ21, AtRS31 and AtSR34) were expressed in the prey vector for yeast two-hybrid assays. As shown in Figure 4A, the C-terminus, but not the N-terminus of MOS14 interacts with the SR proteins. We also tested the interactions between MOS14 and AtRAN1. As shown in Figure 4B, the N-terminus, but not the C-terminus of MOS14 interacts with AtRAN1.

**Figure 5:**
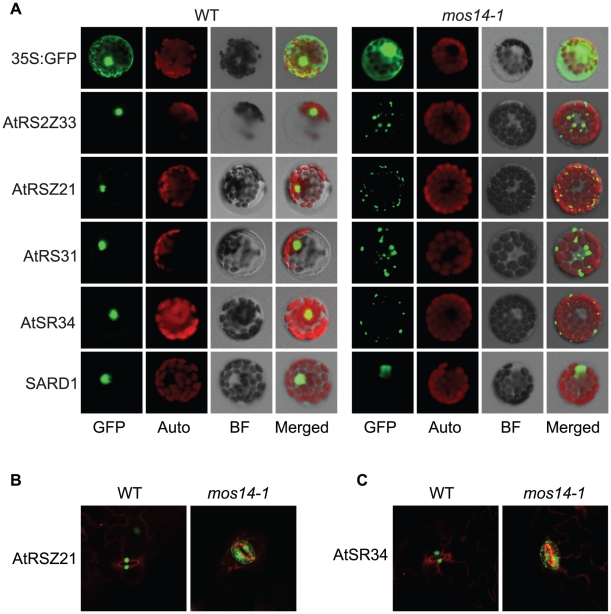
Localization of SR proteins in wild type and *mos14-1*. Localization of SR proteins in wild type and *mos14-1* protoplasts. Protoplasts were prepared from four-week-old soil-grown plants and transformed with 10 µg of plasmid expressing the indicated SR protein with a C-terminal GFP tag or GFP alone . All constructs were under the control of a 35S promoter. GFP (GFP fluorescence); Auto (chloroplast autofluorescence); BF (bright field). (B–C) Localization of AtRSZ21-GFP and AtSR34-GFP in transgenic plants expressing the GFP fusion proteins under the control of 35S promoter in Col-0 (WT) or *mos14-1* background.

To test whether the *mos14-1* mutation affects the nuclear import of Arabidopsis SR proteins, we made constructs expressing four *SR* genes *AtRS2Z33*, *AtRSZ21*, *AtRS31* and *AtSR34* with a C-terminal GFP tag. These constructs were transformed into protoplasts of wild type and *mos14-1* plants to check for the localization of the SR-GFP proteins. A construct expressing the SARD1-GFP fusion protein was included as the control [26]. As shown in Figure 5A, in both wild type and *mos14-1* protoplasts, SARD1 was localized in the nucleus. Consistent with previous studies [27], the SR-GFP proteins were clearly localized in the nucleus of wild type protoplasts. However, in *mos14-1* protoplasts, the SR-GFP proteins were mainly localized in the cytoplasm (Figure 5A and Table 1), suggesting that MOS14 is required for the nuclear localization of SR proteins.

**Table 1:** Analysis of SR protein and SARD1 localization in protoplasts of Col-0 and *mos14-1*.

	Col-0			*mos14-1*		
Targets	nucleus	nucleus+cytoplasm	cytoplasm	nucleus	nucleus+cytoplasm	cytoplasm
AtRS2Z33	98.7%	1.1%	0.3%	1.7%	12.1%	86.2%
AtRSZ21	99.1%	0.9%	—	2.2%	29.2%	68.5%
AtRS31	96.1%	3.3%	0.6%	6.4%	23.4%	70.2%
AtSR34	94.3%	5.3%	0.3%	1.2%	14.6%	84.1%
SARD1	98.7%	0.8%	0.4%	92.9%	3.5%	3.5%

Protoplasts were prepared from four-week-old soil-grown plants and transformed with 10 µg of plasmid expressing the indicated SR protein or SARD1 with a C-terminal GFP tag. All constructs were under the control of a 35S promoter. Percentages of protoplasts with corresponding localization pattern were shown.

Unlike GFP expressed under 35S promoter which is distributed throughout the whole cell, the SR-GFP proteins were localized to discrete foci in the cytoplasm of *mos14-1* protoplasts. The pattern of these foci resembles that of P-bodies, which are distinct foci in the cytoplasm of eukaryotic cells containing many enzymes involved in mRNA turnover. Because of the diverse roles of SR proteins in RNA metabolism, it would not be surprising if they also function in P-bodies. The effect of *mos14-1* on the localization of AtRSZ21 and AtSR34 was further confirmed in transgenic plants expressing the AtRSZ21-GFP and AtSR34-GFP fusion proteins. As shown in Figure 5B and 5C, AtRSZ21-GFP and AtSR34-GFP were localized in discrete foci in the cytoplasm of guard cells in *mos14-1* background. The GFP fusion proteins were also observed in the cytoplasm of leaf pavement cells in *mos14-1*. Taken together, these experiments indicate that MOS14 is a transporter for SR proteins.

### *mos14-1* affects splicing of *SNC1* and *RPS4*

**Figure 6:**
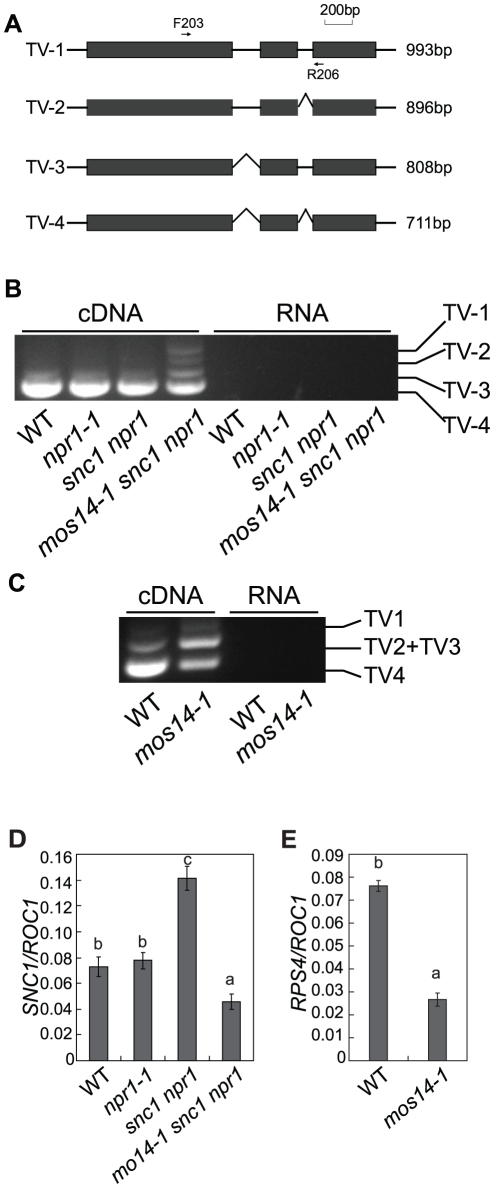
*mos14-1* affects the splicing pattern and causes reduced expression of *SNC1* and *RPS4*. (A) Gene structure of 5′ end of *SNC1*. Exons are indicated with boxes and introns are indicated with lines. Locations of the primers used to amplify the transcript variants (TV) are indicated. The lengths of different transcript variants amplified by F203 and R206 are indicated on the right. (B) Transcription patterns of *SNC1* in wild type (WT), *npr1-1*, *snc1 npr1* and *mos14-1 snc1 npr1*. PCR was performed on cDNA (left) or RNA samples (right) using primer F203 and R206 (Table S1). RNAs incubated in reverse transcription reaction without RTase M-MLV were used as the negative control to ensure that genomic DNA contamination did not occur. (C) *RPS4* alternative transcription patterns in wild type (WT) and *mos14-1*. PCR was performed on cDNA (left) or RNA samples (right). TV1 represents transcripts with both intron 2 and intron 3 retained. TV2 and TV3 represent two transcripts with similar size which retained either intron 2 or intron 3. TV4 is the regular transcript with intron 2 and intron 3 removed. (D) *SNC1* expression in *mos14-1 snc1 npr1*. Values are normalized to the expression of *ROC1*. Bars represent ±SD of three replicates. Statistical differences among the samples are labeled with different letters (*P*<0.01). (E) *RPS4* expression in *mos14-1*. Values are normalized to the expression of *ROC1*. Bars represent ±SD of three replicates. Statistical differences among the samples are labeled with different letters (*P*<0.01).

Multiple *SNC1* transcripts with intron 2 and intron 3 removed or retained have previously been detected [21]. Because none of the transgenic plants expressing the *snc1* cDNA exhibit dwarf morphology like *snc1* mutant plants (Figure S5), alternative splicing is probably required for the function of *SNC1*. Since *mos14-1* affects the nuclear localization of SR proteins and SR proteins participate in pre-mRNA splice site recognition and spliceosome assembly, we tested whether splicing of *SNC1* was affected in *mos14-1*. Primers flanking the introns of *SNC1* were designed to evaluate its splicing pattern of *SNC1* (Figure 6A). Consistent with the previous report, we detected transcripts with either intron 2 or 3 retained (Figure S6). As shown in Figure 6B, we detected another transcript that contains both intron 2 and 3 (TV1) in addition to the regular transcripts with both intron 2 and 3 removed (TV4) in *mos14-1 snc1 npr1*. In wild type plants, the amount of TV2 and TV3 is small compared to that of TV4. Both TV2 and TV3 increased dramatically in the *mos14-1 snc1 npr1* mutant plants (Figure 6B). Similar alteration of *SNC1* transcript patterns was also observed in the *mos14-1* single mutant (Figure S8B). Since PCR reaction using the RNA samples showed no amplification, the DNA fragments from RT-PCR represent *SNC1* transcripts rather than genomic DNA contamination. Further analysis of *SNC1* transcript variants in *mos14-1* and *mos14-1 snc1 npr1* lines carrying the wild type *MOS14* transgene showed that the splicing patterns of *SNC1* in the transgenic lines are similar to those in the wild type plants (Figure S8A and S8B). These data indicate that *mos14-1* affects the splicing of the *SNC1* transcript.

The *R* gene *RPS4* was also reported to be alternatively spliced [23]. We designed primers to detect the transcript variants for *RPS4* by RT-PCR. As shown in Figure 6C, the levels of TV1 are similar in wild type and *mos14-1*. However, TV2+TV3 increased considerably and TV4 was significantly reduced in *mos14-1*, indicating that *mos14-1* also affects the splicing pattern of *RPS4* transcripts. The altered *RPS4* transcript patterns in *mos14-1 snc1 npr1* and *mos14-1* can be complemented by the *MOS14* transgene (Figure S8C and S8D).

To determine whether MOS14 has a general role in RNA splicing, we analyzed splicing of two housekeeping genes *Actin1* and *β-tubulin4* by RT-PCR using primers that flank introns. *ROC1* was used as the control because it contains no intron. We found that splicing of *Actin1* and *β-tubulin4* was not affected in *mos14-1* (Figure S7). We also analyzed the splicing patterns of *U1-70K*, *AtSR30* and *AtSR34*, three genes reported to be alternatively spliced [28], [29]. As shown in Figure S7, the splicing of *AtSR30* and *AtSR34*, but not *U1-70K* was clearly affected by *mos14-1*. Alteration of the transcription patterns of *AtSR30* and *AtSR34* in *mos14-1* further supports the role of MOS14 in alternative splicing. Since the splicing of *Actin1*, *β-tubulin4* and *U1-70K* is not affected by *mos14-1*, there may be a certain level of specificity in MOS14-mediated pre-mRNA processing.

To test whether the splicing defect in *mos14-1* leads to a decrease in *snc1* and *RPS4* transcripts, real-time RT-PCR was carried out using primers to amplify an unspliced region at the 3′ end of the two genes. As shown in Figure 6D and 6E, expression levels of both *snc1* and *RPS4* decreased significantly in the presence of the *mos14-1* mutation.

### *RPS4*-mediated immunity and basal resistance are compromised in *mos14-1*

**Figure 7:**
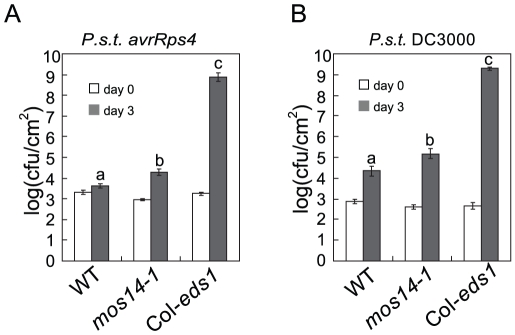
*mos14-1* compromises *RPS*4-mediated immunity and basal resistance. (A) Growth of *P.s.t.* DC3000 *avrRps4* on wild type (WT), *mos14-1* and *eds1-2* (Col). (B) Growth of *P.s.t.* DC3000 on wild type (WT), *mos14-1* and *eds1-2* (Col). Error bars represent ±SD of six replicates. Statistical differences among the samples are labeled with different letters (P<0.01).

Since *mos14-1* altered the splicing pattern of *RPS4* and reduced its expression, we tested whether *RPS4*-mediated immunity is affected by *mos14-1*. As shown in Figure 7A, growth of *Pseudomonas syringae pv. tomato* (*P.s.t.*) DC3000 *avrRps4* in *mos14-1* is about ten-fold higher than that in wild type, suggesting *RPS4*-mediated immunity is compromised in *mos14-1*. We also tested whether MOS14 is required for basal resistance by challenging the *mos14-1* plants with the virulent pathogen *P.s.t.* DC3000. As shown in Figure 7B, bacterial growth is about ten-fold higher in *mos14-1* compared to wild type, indicating that MOS14 is also required for basal resistance.

## Discussion

Previous studies on *snc1* suppressor mutants revealed that multiple components are involved in the regulation of plant immunity. In particular, pathways involved in mRNA export, protein import and protein export were found to contribute to immune responses. Here we report the identification of MOS14 as a novel component of nucleocytoplasmic trafficking required for plant immunity. Loss of *MOS14* function suppresses the constitutive defense responses of *snc1*, compromises resistance mediated by RPS4 and impairs basal resistance against *P.s.t.* DC3000. These findings show that MOS14 plays a critical role in plant immunity.

*MOS14* encodes a nuclear protein with high sequence similarity to TRN-SR proteins in animals. TRN-SR proteins have been shown to function as nuclear import receptors for both phosphorylated SR proteins as well as the splicing repressor protein RSF1 which antagonizes SR proteins in the nucleus [11], [30]. Since their discovery, TRN-SR proteins have not been extensively studied [10]. *MOS14* is a single-copy gene, while the Arabidopsis genome has 18 genes belonging to six subfamilies of SR proteins, of which three are plant-specific [31]. There is no close homolog of *RSF1* in Arabidopsis. Like the TRN-SR proteins in animals, the N-terminus of MOS14 interacts with AtRAN1 and the C-terminus interacts with SR proteins. In addition, localization of several SR proteins to the nucleus was impaired by *mos14-1*. These data indicate that the mechanism of nuclear import of SR proteins is conserved between plants and animals.

Very limited studies have been performed on the genetic characterization of TRN-SR proteins. In *C.elegans*, RNAi of the MOS14 homolog Transporter of SR-1 (TSR-1) leads to embryonic lethality, suggesting TRN-SR proteins can be essential for viability [32]. Intriguingly, the *mos14-1* mutation is not lethal, although it does cause multiple development phenotypes such as reduced stature and fertility. In addition to its functions in development, our genetic analysis of *MOS14* revealed that it plays important roles in both *R* gene-mediated resistance as well as basal defense, suggesting that nuclear import of SR proteins is important for plant immunity. The reasons why *mos14-1* leads to these pleiotropic defects and not lethality awaits further investigation.

SR proteins play important roles in general RNA splicing, alternative splicing, as well as other processes of RNA metabolism. Consistent with the function of MOS14 in the nuclear import of SR proteins, the *mos14-1* mutation affects the splicing of *SNC1* and *RPS4*. Several *R* genes including *SNC1*, *RPS4* and tobacco *N* gene are alternatively spliced, and alternative splicing of *RPS4* and *N* gene are required for their function [23], [33]. In *mos14-1*, alternative splicing of both *SNC1* and *RPS4* are altered. This effect probably contributes to the suppression of *snc1* mutant phenotypes by *mos14-1* and compromised *RPS4* function in the *mos14-1* single mutant. In addition to the altered ratio of transcript variants, the expression levels of *snc1* and *RPS4* were also reduced. The reduced expression of *snc1* and *RPS4* is probably caused by splicing defects resulting from the reduced nuclear localization of SR proteins.

In *mos14-1 snc1 npr1*, the *SNC1* TV-4 transcript level is only modestly reduced, suggesting that reduced accumulation of TV-4 may not be the only factor that contributes to the complete suppression of *snc1* mutant phenotype. In addition to reduced accumulation of TV-4, levels of *SNC1* TV-1, TV-2 and TV-3 are considerably increased in *mos14-1 snc1 npr1*. These transcripts are predicted to produce truncated snc1 proteins because of introduction of early stop codons. It is possible that these truncated proteins may interfere with the function of the full-length snc1. Because *snc1* and *RPS4* are not the only genes whose splicing are affected by *mos14-1*, altered splicing of one or more unknown positive regulators of plant defense could also contribute to the suppression of *snc1* mutant phenotypes.

In addition to the compromised resistance responses mediated by snc1 and RPS4, basal resistance against *P.s.t.* DC3000 is also compromised in *mos14-1*. It remains to be determined how *mos14-1* affects basal resistance. One possibility is that MOS14 is required for the splicing of one or more *R* genes that contribute to basal resistance against *P.s.t.* DC3000. Alternatively, *mos14-1* may cause splicing defects in defense regulators required for basal resistance.

In summary, we have identified MOS14 as a nuclear transporter of SR proteins. Our data suggest that regulation of *R* gene splicing by SR proteins is critical for plant immunity. Future studies on individual SR proteins will help us better understand how SR proteins regulate the splicing of *R* genes.

## Materials and Methods

### Plant growth conditions and mutant screen

All plants were grown at 23°C under 16 hr light/8 hr dark in plant growth rooms or chambers, if not specifically mentioned. To identify mutations that suppress the mutant phenotypes of *snc1*, *snc1 npr1* seeds were mutagenized with EMS. About 30,000 M2 plants representing about 1,500 M1 families were screened for suppression of the dwarf morphology of *snc1 npr1-1*. Mutants lacking the dwarf phenotype were further analyzed for suppression of the constitutive defense responses in *snc1 npr1*.

### Gene expression analysis

About 0.1 g tissue was collected and RNA was extracted by Takara RNAiso reagent. The RNA was treated with Promega RQ1 RNase-Free DNase to remove contaminating genomic DNA. Reverse transcription was subsequently carried out using oligo-dT and the M-MLV RTase cDNA synthesis kit from Takara. About 200 ng of total RNA was included in each RT reaction. For semi-quantitative and real-time PCR , one fiftieth of the cDNA was used in each reaction. A total of 40 cycles were performed for semi-quantitative RCR except 28 cycles for *ROC1*. Real-time PCR was carried out using Takara SYBR® Premix Ex Taq™ II. The primers for real-time PCR analysis of *PR1*, *PR2*, *SNC1* [34] and *ROC1* (also called *cyclophilin*) [35] were described previously. *ROC1* is a housekeeping gene without introns. The sequences of primers used for *SNC1* and *RPS4* transcript variants analysis are shown in Table S1. Primers to amplify *U1-70K* [28], *AtSR30* and *AtSR34* [29] were described previously.

### Pathogen infections and SA measurements

For infections with *H. a.* Noco2, three-week-old soil-grown plants were sprayed with *H. a.* Noco2 at 5×10^4^ spores/ml. The inoculated seedlings were subsequently kept in a growth chamber with high humidity (>80%) at 18°C under 12 hr light/12 hr dark cycle for seven days before growth of *H. a.* Noco2 was quantified, as previously described [36].

For infections with *P.s.t.* DC3000 or *P.s.t.* DC3000 *avrRps4*, five-week-old soil-grown plants were infiltrated with bacterial suspensions (OD_600_ = 0.001) in 10 mM MgCl_2_. Samples were taken at day 0 and day 3.

To analyze the SA levels in the mutant plants, SA was extracted using a previously described procedure [37] and measured by high-performance liquid chromatography.

### Construction of plasmids

For the transgenic complementation test, three PCR fragments, F12R37 (3.9K), F14R38 (3.8K) and F23R19 (2.9K) covering the 11 kb region where *MOS14* is located were amplified from wild type genomic DNA. The primers used for amplification of F12R37, F14R38 and F23R19 are F12, R37, F14, R38, F23 and R19 respectively, and their sequences are provided in Table S1. These fragments were sequentially sub-cloned into pBluescript SK+. The complete 11 kb fragment was subsequently cloned into a modified pGreen0229 vector containing the NOS terminator to obtain the construct *pMOS14:MOS14*. The final construct containing *MOS14* was transformed into *mos14-1 snc1 npr1* through *Agrobacterium*-mediated transformation.

For the subcellular localization study of MOS14, PCR fragments F12R37 (3.9K), F14R38 (3.8K) and F23R20 (2.9K) were sequentially sub-cloned into pBluescript SK+. The primers used for amplification of F23R20 are F23 and R20 and their sequences are listed in the Table S1. The 11 kb fragment described above was cloned into a modified pCambia1305 vector expressing C-terminal tagged *GFP* to obtain *pMOS14:MOS14-GFP*.

For transient expression of SR proteins in protoplasts, full-length cDNAs of *AtRS2Z33*, *AtRSZ21*, *AtRS31* and *AtSR34* without the stop codons were amplified by PCR and cloned into the modified pUC19 vector pUC19-*35S-cmGFP4* that expresses GFP under the 35S promoter.

To obtain transgenic plants expressing *snc1* cDNA, full-length *snc1* cDNA was amplified from total cDNA of snc1 and cloned into a modified pGreen0229 vector. The cDNA clone was sequenced to make sure the sequence is correct and no intron was retained.

To obtain transgenic plants expressing AtSR34-GFP and AtRSZ21-GFP, full-length cDNAs of *AtSR34* and *AtRSZ21* without the stop codons were amplified by PCR and cloned into a modified pCambia1300 vector expressing C-terminal tagged *GFP* under 35S promoter. The constructs were transformed into Col-0 and *mos14-1* through *Agrobacterium*-mediated transformation.

### Yeast two-hybrid analysis

To make constructs for the yeast two hybrid assays, an SfiI restriction site was first introduced to the multiple cloning site of pGBKT7 and pGADT7 to obtain pGBKT7a and pGADT7a, respectively. cDNA expressing the N-terminal or C-terminal region of MOS14 and AtRAN1 were amplified by PCR and cloned into pGBKT7a. Full-length cDNAs of *AtRS2Z33*, *AtRSZ21*, *AtRS31* and *AtSR34* were amplified by PCR and cloned into pGADT7a. cDNA expressing the N-terminal or C-terminal region of MOS14 were also cloned into pGADT7a. The plasmids expressing the MOS14 fragments were co-transformed with the vectors expressing AtRAN1 or one of the SR proteins into the yeast strain PJ694α for yeast two-hybrid analysis.

### Confocal fluorescence microscopy of MOS14-GFP localization

For confocal fluorescence microscopy analysis of MOS14-GFP, the roots or leaves of six-day-old seedling grown on MS plates were first stained with propidium iodide (PI) for 1 min and then washed in ddH_2_O for at least three times. The concentration of PI used for staining the roots was 10 µg/ml, whereas the concentration of PI used for the leaves is 10 mg/ml. The stained sample was observed using a Zeiss Meta 510 confocal microscope. Excitation wavelengths for GFP and PI were 488 nm and 543 nm, respectively. For root samples, the emission filter used for PI was LP560 nm. For leaf samples, the emission filter used for PI was BP560 nm-615 nm. For both root and leaf samples, the emission filter for GFP was BP505 nm-530 nm.

### Confocal fluorescence microscopy of localization of SR-GFPs

Plasmids used for protoplast transfections were purified with Invitrogen PureLink™ HiPure Plasmid Filter Purification Kit. Transformation of wild type or *mos14-1* protoplasts was performed as previously described [38]. After transformation, protoplasts were kept in the dark for about 16 hours. The transformed protoplasts were examined using a Zeiss Axiovert 200 fluorescence microscope. The pictures of representative protoplasts were taken using confocal fluorescence microcopy. For autofluorescence, the emission filter used was 650 nm-740 nm. Confocal fluorescence microscopy analysis of transgenic plants expressing AtSR34-GFP and AtRSZ21-GFP was performed on three-week-old seedlings using a procedure described in the analysis of MOS14-GFP localization.

### Accession numbers

Sequence data from this article can be found in the Arabidopsis Genome Initiative or GenBank/EMBL databases under the following accession numbers: At5g62600 (MOS14), At2g14610 (PR1), At3g57260 (PR2), At4g38470 (ROC1), At2g37620 (Actin1), At5g44340 (β-tubulin4 ), AAD38537 (hTRN-SR1), CAB42634 (hTRN-SR2), NP608708 (dTRN-SR), AF025464 (TSR1) and CAA99366 (MTR10a).

## Supporting Information

Figure S1 Alignment of *MOS14* wild type cDNA and the cDNA variants from *mos14-1*. Red asterisk indicates the site of the point mutation in *mos14-1*.
(PDF)

Figure S2 Morphology of five-week-old soil-grown plants of Col-0 (WT), *mos14-1*, and *mos14-1* carrying the *MOS14* transgene.
(PDF)

Figure S3 Alignment of MOS14 and transportin-SRs from other eukaryotes. Amino acid sequences of hTRN-SR1 and hTRN-SR2 from human, dTRN-SR from *Drosophila*, TSR1 from *C.elegans*, MTR10a from *S.cerevisiae* and MOS14 were aligned by the CLUSTALW program (http://www.ebi.ac.uk/Tools/clustalw2/index.html) and the multiple sequence alignment result was further analyzed by the BOXSHADE software (http://www.ch.embnet.org/software/BOX_form.html). The importin β N-terminal domain is underlined.
(PDF)

Figure S4 Morphology of five-week-old soil-grown plants of Col-0 (WT), *mos14-1*, and *mos14-1* carrying the *MOS14-GFP* transgene.
(PDF)

Figure S5 Morphology of three-week-old Col-0 (WT), *snc1*, and three representative T1 transgenic plants expressing the *snc1* cDNA under 35S promoter in Col-0 wild type background.
(PDF)

Figure S6 Analysis of alternative transcripts of *SNC1* in wild type (WT), *npr1-1*, *snc1 npr1* and *mos14-1 snc1 npr1*. (A) Gene structure of 5′ end of *SNC1*. Exons are indicated with boxes and introns are indicated with lines. Locations of the primers used to amplify the transcript variants (TV) are indicated. (B) Transcription patterns of *SNC1* in wild type (WT), *npr1-1*, *snc1 npr1* and *mos14-1 snc1 npr1*. PCR was performed on DNase I-treated total RNA. RNAs incubated in reverse transcription reaction without RTase M-MLV were used as the negative control to ensure that genomic DNA contamination did not occur. Primers F203 and R204 were used to detect transcripts with or without the second intron (upper panel). Primers F205 and R206 were used to detect transcripts with or without the third intron (lower panel). Primers are listed in Table S1.
(PDF)

Figure S7 Analysis of transcripts of *Actin1*, *β-tubulin4*, *U1-70K*, *AtSR30*, *AtSR34* and *ROC1* in wild type (WT) and *mos14-1*. Primers used to amplify *Actin1* and *β-tubulin4* are listed in Table S1.
(PDF)

Figure S8 Reverse of *SNC1* and *RPS4* splicing patterns in *mos14-1* and *mos14-1 snc1 npr1* by the *MOS14* transgene. (A–B) *SNC1* splicing patterns in the indicated genotypes. Primers used were F203 and R206. (C–D) *RPS4* splicing patterns in the indicated genotypes. Primers used to amplified *RPS4* are listed in Table S1. “*MOS14g*” stands for “*mos14-1 snc1 npr1* containing the *MOS14* transgene under its native promoter”.
(PDF)

Table S1 Primers used in this work.
(DOC)
