# Supplementary material for: Transportin-SR Is Required for Proper Splicing of Resistance Genes and Plant Immunity
Source: PLoS Genet. 2011 Jun 30;7(6):e1002159. doi: 10.1371/journal.pgen.1002159 (PMC3128105; doi:10.1371/journal.pgen.1002159)
Supplement: Figure S1 — Alignment of MOS14 wild type cDNA and the cDNA variants from mos14-1. Red asterisk indicates the site of the point mutation in mos14-1. (PDF) [file pgen.1002159.s001.pdf]

```
cDNA    AACCGAAGACTGCATATCTTTCAGCCAGCTTATCAGAGCCTTGTATCTTTGGTTGGCTTC 60
TV1     AACCGAAGACTGCATATCTTTCAGCCAGCTTATCAGAGCCTTGTATCTTTGGTTGGCTTC 60
TV2     AACCGAAGACTGCATATCTTTCAGCCAGCTTATCAGAGCCTTGTATCTTTGGTTGGCTTC 60
TV3     AACCGAAGACTGCATATCTTTCAGCCAGCTTATCAGAGCCTTGTATCTTTGGTTGGCTTC 60
TV4     AACCGAAGACTGCATATCTTTCAGCCAGCTTATCAGAGCCTTGTATCTTTGGTTGGCTTC 60
TV5     AACCGAAGACTGCATATCTTTCAGCCAGCTTATCAGAGCCTTGTATCTTTG--------- 51
TV6     AACCGAAGACTGCATATCTTTCAGCCAGCTTATCAGAGCCTTGTATCTTTG--------- 51

cDNA    AGAGTTCAGTATCCTGAAGATTATCAAGGCCTCTCATATGAGGACCTTAAGGAATTCAAG 120
TV1     AGAGTTCAGTATCCTGAAGATTATCAAGGCCTCTCATATGAGGACCTTAAGGAATTCAAG 120
TV2     AGAGTTCAGTATCCTGAAGATTATCAAGGCCTCTCATATGAGGACCTTAAGGAATTCAAG 120
TV3     AGAGTTCAGTATCCTGAAGATTATCAAGGCCTCTCATATGAGGACCTTAAGGAATTCAAG 120
TV4     AGAGTTCAGTATCCTGAAGATTATCAAGGCCTCTCATATGAGGACCTTAAGGAATTCAAG 120
TV5     ------------------------------------------------------------ 51
TV6     ------------------------------------------------------------ 51

                    *
cDNA    CAGACTAGATATG----------------------------------------------- 133
TV1     CAGACTAGATATAGTAATCAAAGCGGTGTATTACTTGTTCTCAATATTACATTAGATCAA 180
TV2     CAGACTAGATATAGTAATCAAAGCG----------------------------------- 145
TV3     CAGACTAGATATA----------------------------------------------- 133
TV4     CAGACTAGATATAGTAATCAAAGCG----------------------------------- 145
TV5     ------------------------------------------------------------ 51
TV6     ------------------------------------------------------------ 51

cDNA    ----------CTGTTGCAGATGTATTAATAGATGCAGCGTTAATCCTGGGAGGGGATACT 183
TV1     GATCTTTTGA---------ATGTATTAATAGATGCAGCGTTAATCCTGGGAGGGGATACT 231
TV2     -------------------ATGTATTAATAGATGCAGCGTTAATCCTGGGAGGGGATACT 186
TV3     -------------------ATGTATTAATAGATGCAGCGTTAATCCTGGGAGGGGATACT 174
TV4     -------------------------------ATGCAGCGTTAATCCTGGGAGGGGATACT 174
TV5     ----------CTGTTGCAGATGTATTAATAGATGCAGCGTTAATCCTGGGAGGGGATACT 101
TV6     -------------------ATGTATTAATAGATGCAGCGTTAATCCTGGGAGGGGATACT 92
```
