# Supplementary figures and images for: Transportin-SR Is Required for Proper Splicing of Resistance Genes and Plant Immunity
Source: PLoS Genet. 2011 Jun 30;7(6):e1002159. doi: 10.1371/journal.pgen.1002159 (PMC3128105; doi:10.1371/journal.pgen.1002159)

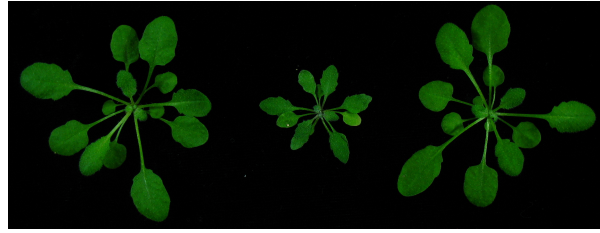

WT

*mos14-1*

*pMOS14:MOS14 in mos14-1*

Supplement: Figure S2 — Morphology of five-week-old soil-grown plants of Col-0 (WT), mos14-1, and mos14-1 carrying the MOS14 transgene. (PDF) [file pgen.1002159.s002.pdf]

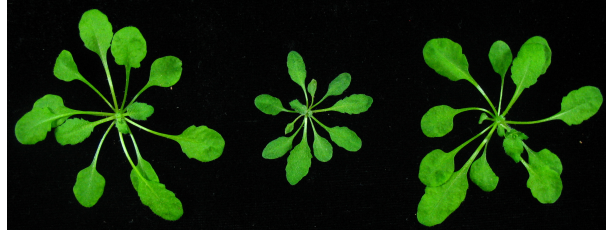

WT

*mos14-1*

pMOS14:MOS14-GFP in *mos14-1*

Supplement: Figure S4 — Morphology of five-week-old soil-grown plants of Col-0 (WT), mos14-1, and mos14-1 carrying the MOS14-GFP transgene. (PDF) [file pgen.1002159.s004.pdf]

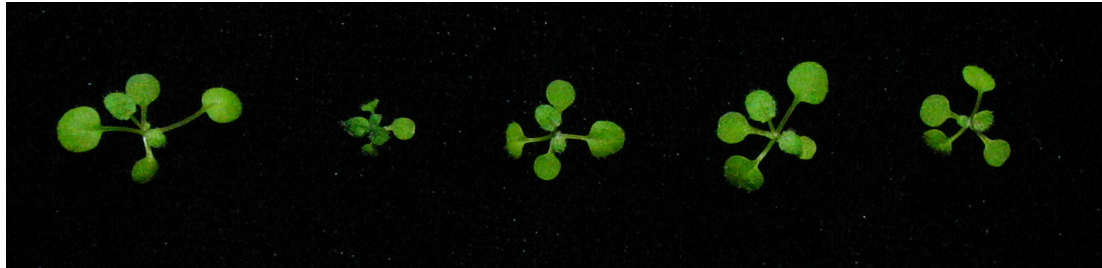

WT

snc1

35S:snc1 #1

35S:snc1 #2

35S:snc1 #3

Supplement: Figure S5 — Morphology of three-week-old Col-0 (WT), snc1, and three representative T1 transgenic plants expressing the snc1 cDNA under 35S promoter in Col-0 wild type background. (PDF) [file pgen.1002159.s005.pdf]

**A**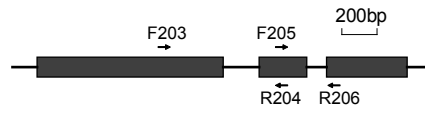**B**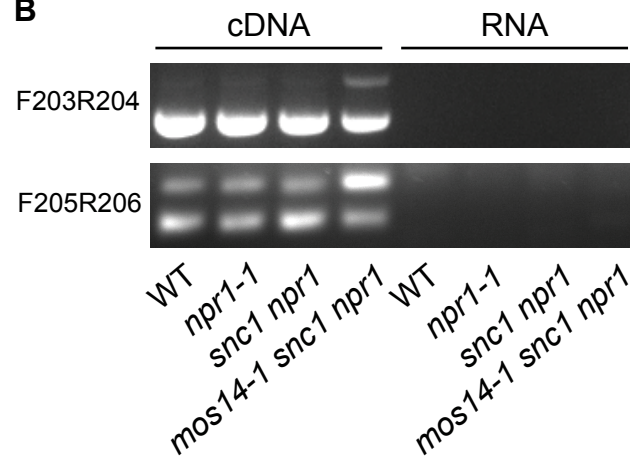

Supplement: Figure S6 — Analysis of alternative transcripts of SNC1 in wild type (WT), npr1-1, snc1 npr1 and mos14-1 snc1 npr1. (A) Gene structure of 5′ end of SNC1. Exons are indicated with boxes and introns are indicated with lines. Locations of the primers used to amplify the transcript variants (TV) are indicated. (B) Transcription patterns of SNC1 in wild type (WT), npr1-1, snc1 npr1 and mos14-1 snc1 npr1. PCR was performed on DNase I-treated total RNA. RNAs incubated in reverse transcription reaction without RTase M-MLV were used as the negative control to ensure that genomic DNA contamination did not occur. Primers F203 and R204 were used to detect transcripts with or without the second intron (upper panel). Primers F205 and R206 were used to detect transcripts with or without the third intron (lower panel). Primers are listed in Table S1. (PDF) [file pgen.1002159.s006.pdf]

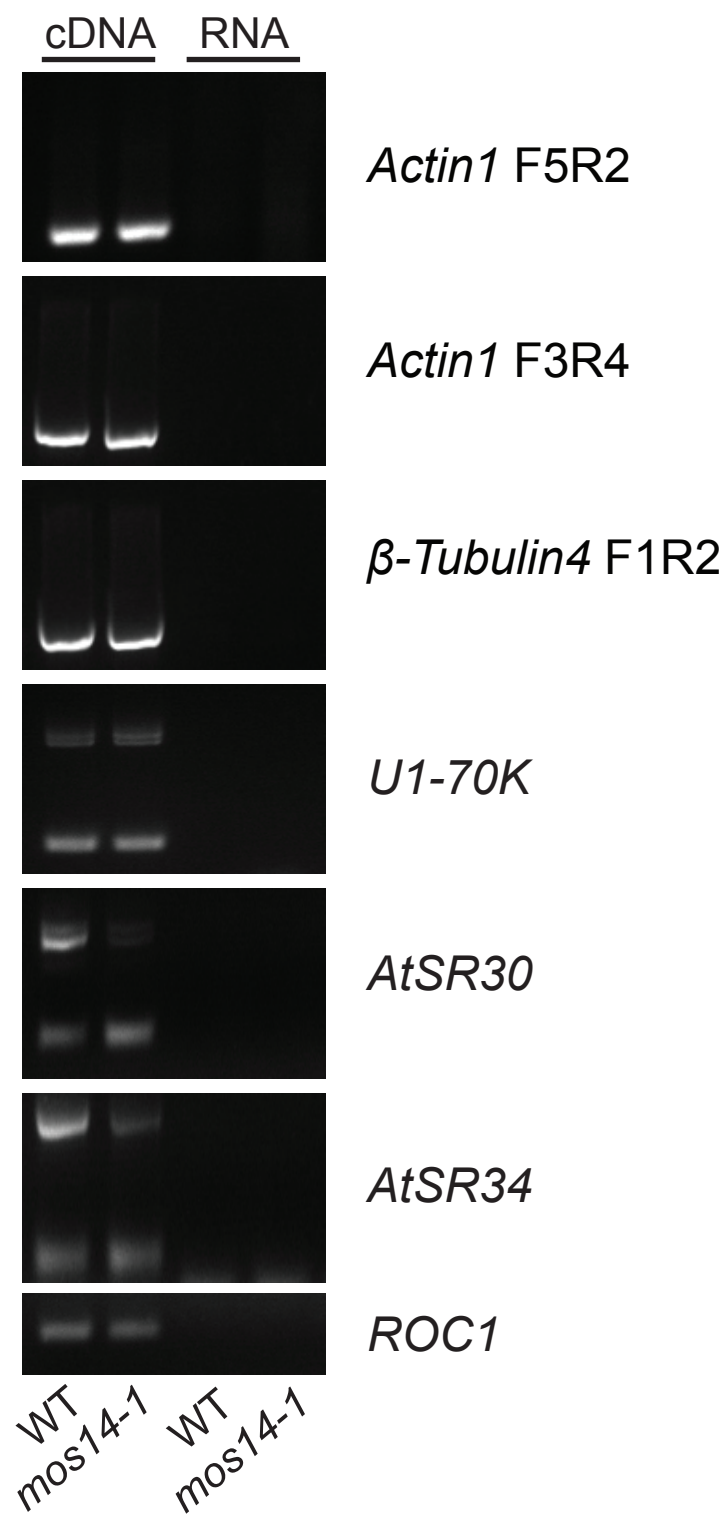

Supplement: Figure S7 — Analysis of transcripts of Actin1, β-tubulin4, U1-70K, AtSR30, AtSR34 and ROC1 in wild type (WT) and mos14-1. Primers used to amplify Actin1 and β-tubulin4 are listed in Table S1. (PDF) [file pgen.1002159.s007.pdf]

**A**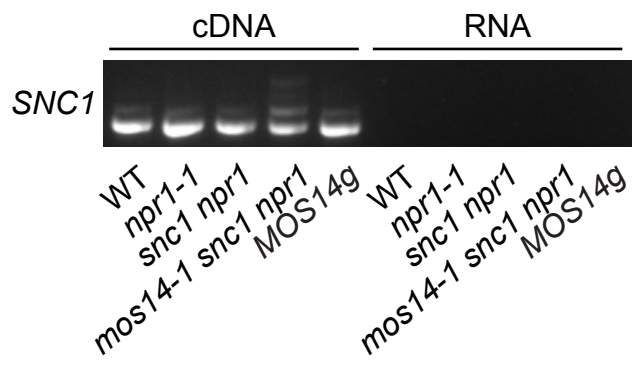**B**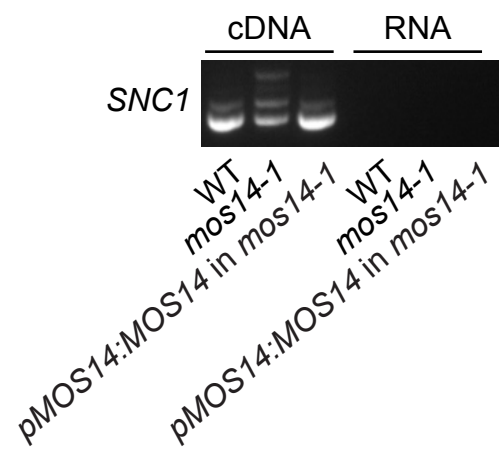**C**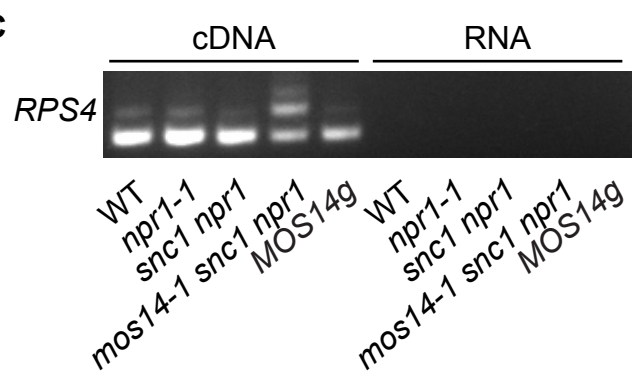**D**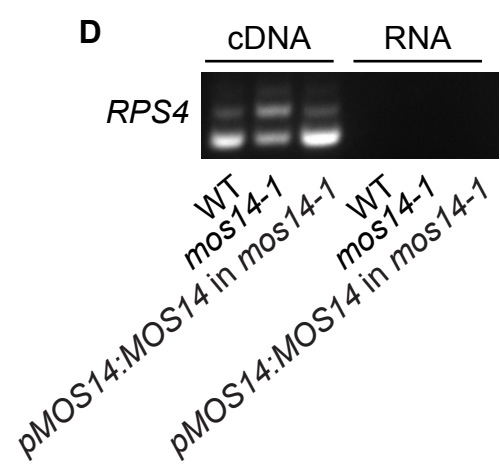

Supplement: Figure S8 — Reverse of SNC1 and RPS4 splicing patterns in mos14-1 and mos14-1 snc1 npr1 by the MOS14 transgene. (A–B) SNC1 splicing patterns in the indicated genotypes. Primers used were F203 and R206. (C–D) RPS4 splicing patterns in the indicated genotypes. Primers used to amplified RPS4 are listed in Table S1. “MOS14g” stands for “mos14-1 snc1 npr1 containing the MOS14 transgene under its native promoter”. (PDF) [file pgen.1002159.s008.pdf]
