# Supplementary material for: Transportin-SR Is Required for Proper Splicing of Resistance Genes and Plant Immunity
Source: PLoS Genet. 2011 Jun 30;7(6):e1002159. doi: 10.1371/journal.pgen.1002159 (PMC3128105; doi:10.1371/journal.pgen.1002159)
Supplement: Figure S3 — Alignment of MOS14 and transportin-SRs from other eukaryotes. Amino acid sequences of hTRN-SR1 and hTRN-SR2 from human, dTRN-SR from Drosophila, TSR1 from C.elegans, MTR10a from S.cerevisiae and MOS14 were aligned by the CLUSTALW program (http://www.ebi.ac.uk/Tools/clustalw2/index.html) and the multiple sequence alignment result was further analyzed by the BOXSHADE software (http://www.ch.embnet.org/software/BOX_form.html). The importin β N-terminal domain is underlined. (PDF) [file pgen.1002159.s003.pdf]

```
MOS14       1 ----MEHQNAVKEALNALYHHPDDTVRVHADRWLQNFQGTLDAWQVADNLLHDSSS---N
hTRN-SR1    1 MEGAKPTLQLVYQAVQALYHDPDPSGKERASFWLGELQRSVHAWEISDQLLQIRQ----D
hTRN-SR2    1 MEGAKPTLQLVYQAVQALYHDPDPSGKERASFWLGELQRSVHAWEISDQLLQIRQ----D
dTRN-SR     1 MD--TYSVDVVYQAISALFQGNNPKEQEKANKWLQDFQKSIYSWTIADELLHQKR----D
TSR1        1 --------------------------MGKPFFFFGNFGLKNPTICAIEHICHRGC----G
MTR10a      1 --MDNLQVSDIETALQCISSTASQDDKNKALQFLEQFQRSTVAWSICNEILSKEDPTNAL

MOS14      54 LETLIFCSQTLRSKVQRDFEELPPGAFQKLRQSLTTLLKKFHKGPP-KVRTQISIAVAAL
hTRN-SR1   57 VESCYFAAQTMKMKIQTSFYELPTDSHASLWDSLLTHIQNLKDLSP-VIVTQLALAIADL
hTRN-SR2   57 VESCYFAAQTMKMKIQTSFYELPTDSHASLRDSLLTHIQNLKDLSP-VIVTQLALAIADL
dTRN-SR    55 LHANYFAAQTMRNKIQNSFSELPPHTHESLRDSLITHIGQIDEQTDNVIVTQLSLAVADL
TSR1       31 SYAQIFIFHSKTCNFFHFFKFFCSRHGASAHDAQSEATS-----------TQLCLAIADL
MTR10a     59 LELNIFAAQTLRNKVTYDLSQLEN-NLPQFKDSLLTLLLSHN---QKLIITQLNVALARL

MOS14     113 AVHVPAADWGDGGIISWLRDEMHMHPEYVPGFLELLTVLPEETFNYKIAARPDRRR--QF
hTRN-SR1  116 ALQMPS--WK--GCVQTLVEKYSNDVTSLPFLLEILTVLPEEVHSRSLRIGANRRT--EI
hTRN-SR2  116 ALQMPS--WK--GCVQTLVEKYSNDVTSLPFLLEILTVLPEEVHSRSLRIGANRRT--EI
dTRN-SR   115 ALLMAS--WR--EPINDLLVTLAPHQCAIWPLLEVLKVLPEEIDSRYLRLGANRRE--EV
TSR1       80 YIQVPT--WNN-WIFELLNQCQALEGDRTVMTLTLLQVFPEEVEQIRG-VGENRRH--AI
MTR10a    115 AIQFLE--WQN-----PIFEIISLLNSSPSILLNFLRILPEETLDIASTSLTEVEFNSRI

MOS14     171 EKELTSQMEAALSILSACLKISELK----------EQVLEAFASWLRLRHGIPGTVLACH
hTRN-SR1  170 IEDLAFYSSTVVSLLMTCVEKAGTD------EKMLMKVFRCLGSWFNL-GVLDSNFMANN
hTRN-SR2  170 IEDLAFYSSTVVSLLMTCVEKAGTD------EKMLMKVFRCLGSWFNL-GVLDSNFMANN
dTRN-SR   169 HKQLDASAECVLKFLCMCLQREDLDQ-----QRVWNAALRTYSAWLVI-HAFPVSHVYNN
TSR1      134 RNELAACEQPMITFLSHVLEKFHSN------ADVLKRVFKCLESNLQN-HQMRTDHFAAS
MTR10a    168 HELIDPIAEDVLKFLVSCIDLLQNTDGNSSSSISLEQILRCLNSWSYE--FPVEQLLTVQ

MOS14     221 PLVHAALSSLN------CDPLSEASVNVISELIHHTASPS-SGGISAQTPLIQVIVPQIL
hTRN-SR1  223 KLLALLFEVLQQDK--TSSNLHEAASDCVCSALYAIEN------VETNLPLAMQLFQGVL
hTRN-SR2  223 KLLALLFEVLQQDK--TSSNLHEAASDCVCSALYAIEN------VETNLPLAMQLFQGVL
dTRN-SR   223 ALTQLAFRLLSLPE--TSGKLHDNATECVCALLSCINTRQ-DGASDPESSFEAQLFGAVC
TSR1      187 PLISMIFHVIASIDPIIPSCLHETATNCIVQALYRVED------LEMHRKLAEVVHRGVI
MTR10a    226 PLINLVFETISNGNE-SDMEAFDSAIDCLCVILRESRDTTNEQLISALFHQLMLLQEKLL

MOS14     274 SLQAHLRDSSKDEEDVKAIG--RLFADVGDSYVELIATGSDE------PMVIVHALLEVT
hTRN-SR1  275 TLETAYHMAVAREDLDKVLNYCRIFTELCETFLEKIVCTPGQG---LGDLRTLELLLICA
hTRN-SR2  275 TLETAYHMAVAREDLDKVLNYCRIFTELCETFLEKIVCTPGQG---LGDLRTLELLLICA
dTRN-SR   280 MLETPYHLSVAHEDTDKTINYCRIFTSLCDAFFYDLLADAQKP---HYSLKGLDLVLLCV
TSR1      241 SLVSAFEKAQQVEDFDRLQNIARIFVEMVESFYVQIVNEANPDPAAIGSLACFELLLLVA
MTR10a    285 PTLFTDHPLNDEYDDDLLEGMTRLFVEAGEAWSVVISKNPDFF-----KPMVLVLLMLTC

MOS14     326 AHPEF---------DIASMTFNFWHSLQLMLTKRESYSSLGSEASIEVERNRRLHIFQPA
hTRN-SR1  332 GHPQY---------EVVEISFNFWYRLGEHLYKTN--------------DEVIHGIFKAY
hTRN-SR2  332 GHPQY---------EVVEISFNFWYRLGEHLYKTN--------------DEVIHGIFKAY
dTRN-SR   337 GHFDY---------EVAEITFHLWYKLSEDLFQRN--------------EDKLTVLFRPH
TSR1      301 KHHDWALKIAIFLLQLIEMSFNVWYRITEELFKYD--------------DDQYIGRFRPY
MTR10a    340 KNEDL---------DVVSYTFPFWFNFKQSLVLPR--------------YQESRKAYSDI

MOS14     377 YQSLVSLVGFRVQYPEDYQGLSYEDLKEFKQTRYAVADVLIDAALILGGDTTLKILYMKL
hTRN-SR1  369 IQRLLHALARHCQLEPDHEGVP-EETDDFGEFRMRVSDLVKDLIFLIGSMECFAQLYSTL
hTRN-SR2  369 IQRLLHALARHCQLEPDHEGVP-EETDDFGEFRMRVSDLVKDLIFLIGSMECFAQLYSTL
dTRN-SR   374 IERLISALFRHSQMESDHDGLI-EENNNFFDFRRKVSDLIKDVAFIVGSGACFKQMFHIL
TSR1      347 AEKFIQCLYEHCKLDADDVDDLLDETSEFGEFRLKAVEALRDVVFIVNSDKCIQMMHQKL
MTR10a    377 FVKLINGIITHLQYPSGQFSSK-EEEDKFKDFRYHMGDVLKDCTAVVGTSEALSQPLIRI

MOS14     437 LEANAQTGNNFQDWRPAEAILFCIWAISNYVSV---------------------------
hTRN-SR1  428 KEGNPP-------WEVTEAVLFIMAAIAKSVDPKKPFSNAACHHSLLFGQNITSEISNCE
hTRN-SR2  428 KEGNPP-------WEVTEAVLFIMAAIAKSVDP---------------------------
dTRN-SR   433 QAPETT-------WESTEAALFIMQNVAKNILP---------------------------
TSR1      407 IECCHKPN---ASWEESESALFVMSAVVQNLLP---------------------------
MTR10a    436 KSAIENNN----SWQIMEAPLFSLRTMAKEISL---------------------------
```
