# Supplementary material for: Transportin-SR Is Required for Proper Splicing of Resistance Genes and Plant Immunity
Source: PLoS Genet. 2011 Jun 30;7(6):e1002159. doi: 10.1371/journal.pgen.1002159 (PMC3128105; doi:10.1371/journal.pgen.1002159)
Supplement: Table S1 — Primers used in this work. (DOC) [file pgen.1002159.s009.doc]

**Table S1. Primers used in this work**

| primer | 5'-3' sequence | purpose |
| --- | --- | --- |
| ROC1 real-F | gtctgatagagatctcacgt | for real time |
| ROC1 real-R | ccctggcacatgaagttcg | for real time |
| RPS4 real-F | gctagaccatgtcttcattgga | for real time |
| RPS4 real-R | tcttttcaacaggcataacgtac | for real time |
| MOS14-F47 | tcgattattcgctgacgtag | mos14 KO |
| MOS14-SNPR | taagagtagtatcccctccc | mos14 KO |
| SNC1-F203 | agggaaggaccaaagaatgg | alternative splicing |
| SNC1-R204 | tgaggtagatccccgtaata | alternative splicing |
| SNC1-F205 | aaattggttattacggggatc | alternative splicing |
| SNC1-R206 | aattgttggaatacctcaaatt | alternative splicing |
| RPS4-AS-F1 | ctgtggctccatcaacacat | alternative splicing |
| RPS4-AS-R2 | gatcgacccaccttaagcat | alternative splicing |
| ACTIN1 AS-R2 | tgagagttaaaatacctcgt | alternative splicing |
| ACTIN1 AS-F3 | tgagactttcaatgcccct g | alternative splicing |
| ACTIN1 AS-R4 | ctgaaaaatgatcaactttgaac | alternative splicing |
| ACTIN1 AS-F5 | acattcaacctcttgtttgc | alternative splicing |
| βTUB 4 AS-F1 | tacgtcggcgattctccgtt | alternative splicing |
| βTUB4 AS-R2 | gggtcagttcaggaacactc | alternative splicing |
| MOS14-F12 | ctgctcgtccggatcgacg | complementary/GFP |
| MOS14-R37 | cgcggatcccagacctttcgactagctgcc | complementary/GFP |
| MOS14-F14 | cggggtaccagaggacaagctactgaccg | complementary/GFP |
| MOS14-R38 | agcatacccatgcctaagac | complementary/GFP |
| MOS14-F23 | tgaagctgaagtgatgcccc | complementary/GFP |
| MOS14-R19 | cgcggatccttatgatacaggagcagtaacca | complementary |
| MOS14-R20 | cgcggatcctgatacaggagcagtaacca | GFP |
| MMN10-F | agctgcaataatgccaaagg | InDel marker |
| MMN10-R | gaaccatcaccactggtgag | InDel marker |
| MUB3-F | aatagatcaaagcctggctg | InDel marker |
| MUB3-R | gattcctttgcttaccacac | InDel marker |
| K22G18-F | gtgtagtttttgcctttctag | SNP marker |
| K22G18-col R | cgattgaatctcaagcaacg | SNP marker |
| K22G18-ler R | cgattgaatctcaagcaacc | SNP marker |
| MQB2-F | actaaaaggcgactactagc | InDel marker |
| MQB2-R | ttgccatttatttggtcaacac | InDel marker |
| MMI9-col F | gaaagctatcagtataattgtataaag | SNP marker |
| MMI9-ler F | ttgaaacctatcagtataatgatatc | SNP marker |
| MMI9-R | ccgagaccggtttgaattgc | SNP marker |
| K19B1-seq F | tagcaacttacaccacgcag | sequence marker |
| K19B1-seq R | aagagtaaatctaattggtcgg | sequence marker |
| MRG21-seq NF | ctcgtgtaatcaagacaacc | sequence marker |
| MRG21-seq NR | ttcatcatccctagatctcc | sequence marker |
| SNC1 cDNAF | cggggtaccatgatggatacatccaaagatgatgatatggagatagcttcttc | 35S |
| SNC1 cDNAR | cgcggatcctcagttaccagaaacaggaaac | 35S |
